# Target Trial Emulation: Impact of C-Section on Birth Outcomes for Spontaneous Preterm Breech Presentations

**DOI:** 10.23889/ijpds.v9i5.2785

**Published:** 2024-09-18

**Authors:** Robin Alexander, Holger Unger, Swetha Bhaskar, Ben Swallow, Stefan Unger, Amaya Azcoaga-Lorenzo, Adeniyi Fagbamigbe, Sarah Stock, Colin McCowan

**Affiliations:** 1 University of St Andrews; 2 Liverpool School of Tropical Medicine; 3 Charles Darwin University; 4 Victoria Hospital, NHS Fife; 5 University of Edinburgh; 6 Royal Hospital for Sick Children; 7 Instituto de Investigación Sanitaria Fundación Jimenez Diaz; 8 University of Ibadan

## Objective

In 2023 a public panel around public sector data use in Scotland was refreshed. The aim was to have a panel not only feedback on individual research projects but also the broader systems and processes of the data landscape in Scotland – from data sourcing and data storage, to data sharing and communicating research findings.

## Approach

The public panel is a group of c. twenty members of the public and has, to date, focused on:

Recruiting a representative sample of people
Developing training materials to cover all aspects of the data system
Building mechanisms to incorporate feedback into system change
Increasing transparency of the panel discussions to support wider sector (including enhancing our website content and working to develop an annual report)
Embedding findings from discussions with the public panel across the system in Scotland

## Result

Feedback from panel members on recruitment and our approach has been positive. Panel discussions have already influenced ongoing work including synthetic data, website content and research projects. The panel have also begun advising on a system wide change from ‘just in time’ to ‘research ready’ data sets. This work followed a review of current data approvals processes across Scottish Safe Havens and the UK after COVID-19.

## Conclusions and Implications

The current panel membership will run for two years after which a review of approach and impact will be carried out. Increasing transparency of the findings from the panels will enable organisations across the sector to benefit from this work.

